# A case report of Tumor-Induced Osteomalacia (TIO) caused by central giant cell granuloma

**DOI:** 10.1016/j.bonr.2024.101804

**Published:** 2024-09-10

**Authors:** Sevil Ghaffarzadeh Rad, Amir Bahrami, Jalil Houshyar, Nazila Farrin, Farzad Najafipour, Halimeh Amirazad

**Affiliations:** a Endocrine Research Center, Tabriz University of Medical Sciences, Tabriz, Iran; b Nutrition Research Center, Tabriz University of Medical Sciences, Tabriz, Iran

**Keywords:** Osteomalacia, Hypophosphatemia, Metabolic bone disease, Osteoporosis

## Abstract

**Introduction:**

Tumor-induced osteomalacia (TIO) is a rare paraneoplastic syndrome defined by severe hypophosphatemia, bone loss, fractures, and muscle weakness. Identifying of the tumor site is often difficult. The primary treatment for Tumor-induced osteomalacia (TIO) is currently surgical resection. Removing the primary tumor is the most definitive treatment for this disease.

**Methods:**

Here we describe the case of a 32-year-old man who exhibited sever muscle weakness and pain that had continued for three years. The patient has three sisters and one brother, all of whom are completely healthy and free of bone and muscle problems.

Laboratory data indicate low serum phosphorus, normal serum and urine calcium level, besides raised alkaline phosphatase level. Due to elevated phosphorus levels in the urine and the lack of an alternative source for phosphorus excretion, along with the absence of short stature, bone deformities, and a negative family history that might suggest the potential for Tumor-induced osteomalacia (TIO), an octreotide scan was performed to the localized the tumor site. The scan, corroborated by CT and MRI scans, displayed absorption in the right maxillary sinus. Surgical excision of the lesion confirmed it to be a central giant cell granuloma.

**Results:**

Following surgery and without receiving any other treatment, the patient's phosphorus levels and clinical condition improved compared to before the surgical treatment. Subsequently, the symptoms of muscle weakness and skeletal pain significantly diminished, and the patient regained the ability to move.

**Conclusion:**

Tumor enucleation was conducted, and the pathological examination of the maxillary sinus lesion unveiled a central Giant cell granuloma. The patient had clinical and laboratory improvement after surgery. This finding confirmed our diagnosis of a paraneoplastic hypophosphatemia associated with a giant cell granuloma.

## Highlights

• TIO is a paraneoplastic syndrome characterized by hypophosphatemia, muscle weakness and bone fractures.
• The first and the most definitive treatment for TIO currently is surgical resection.
• The challenge of locating the tumor can cause additional delays in treatment.
• In this case, we identified the lesion using functional imaging, followed by CT scan and MRI.
• Following surgical resection, the patient's phosphorus levels and clinical condition improved.

## 1 Introduction

Tumor-induced osteomalacia (TIO) is an uncommon paraneoplastic syndrome caused by small, benign mesenchymal tumors that secrete the phosphaturic hormone, fibroblast growth factor 23 (FGF23), leading to hypophosphatemia and skeletal pain, fractures and muscle fatigue. FGF23 causes hypophosphatemia and osteomalacia by reducing phosphate reabsorption in proximal tubules and inhibiting 25-hydroxyvitamin D reabsorption by inhibiting 1α-hydroxylation (Yin et al., 2018; Dahir et al., 2021). Hypophosphatemia is usually severing and marked by musculoskeletal discomfort, and weakness in proximal muscles, waddling gait, multiple fractures, and rarely local symptoms (Feng et al., 2017; De Beur, 2005). If left untreated can cause secondary/tertiary hyperparathyroidism, hypercalciuria, and nephrocalcinosis. TIO is typically caused by mesenchymal tumor, characteristically small and benign tumors that can be placed in anyplace in the body specifically in soft tissue or bone. Due to a limited comprehension of the disease, there is a high likelihood of either misdiagnosis or clinical oversight (Zhang et al., 2023). It is an uncommon condition, with nearly 1000 cases reported globally up to now. The exact prevalence is unidentified due to the lack of population-based epidemiologic studies (Brandi et al., 2021). The symptoms and manifestation of TIO do not uniquely indicate this condition and indeed, patients often wait at least 2.5 to 3 years to obtain a precise diagnosis (Dahir et al., 2021). The challenge of locating the tumor can cause additional delays in treatment. The difficulty in diagnosing the tumor's location is a significant aspect of this challenge (Dahir et al., 2021) (Feng et al., 2017). The main and curative treatment is surgical resection of tumor if it can be found. If the tumor's location is not identified, vitamin D and phosphorus supplements are used (Jadhav et al., 2023). Novel treatments include the use of monoclonal antibodies like borusumab (Aligail et al., 2022). Treatment typically involves a combination of vitamin D (calcitriol and alfacalcidol) and phosphate supplementation. In cases where a confirmed mesenchymal tumor consistent with TIO is found, surgical removal is advised, offering a potential cure (Bosman et al., 2022).

## 2 Case report

We report an uncommon case of hypophosphatemic osteomalacia presenting with significant osteoporosis and bilateral femoral fractures due to tumor-induced osteomalacia associated with phosphorus deficiency. Histological examination of an enucleated tumor revealed a central giant cell granuloma. A 32-year-old man was referred to the endocrine clinic due to muscle weakness and pain. The patient had been healthy until three years ago, but since then, he has complained of back and hip pain, as well as progressively disabling weakness.

**Fig. 1:**
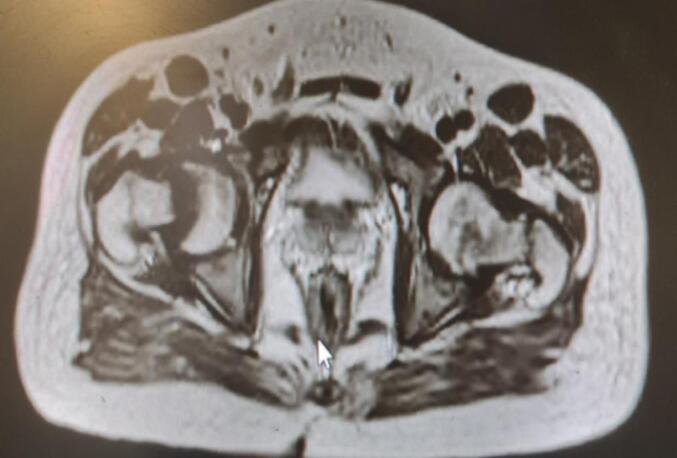
MRI of Bilateral femoral neck fracture with slipped right femoral head and AVN in right femoral.

Upon examination, the patient did not have short stature and showed no dental problems or bone and skeletal deformities. In the last six months, his muscle fatigue progressed in severity, leading to six months of bed confinement before admission. Brain MRI and EMG/NCV tests performed by a neurologist revealed normal results. He was referred to the endocrine clinic due to hypophosphatemia. Radiographs showed significant osteoporosis. BMD measurements showed the following results: In the lumbar region, the T-score was ‐3.4, with a corresponding *Z*-score of ‐3.4. In the femur region, the T-score was ‐2.6, and the *Z*-score was ‐2.4. Additionally, in the distal radius, the T-score was ‐2.7, with a corresponding Z-score of ‐2.8. Furthermore, lumbar radiography, revealed a slight decrease in the height of the vertebrae, along with bilateral femoral head fracture and stage D avascular necrosis on the right femur (Fig. 1). Click here to access the complete DICOM file. https://www.dicomlibrary.com?study=1.3.6.1.4.1.44316.6.102.1.20240608232617351.98565746511053485969.

**Fig. 2:**
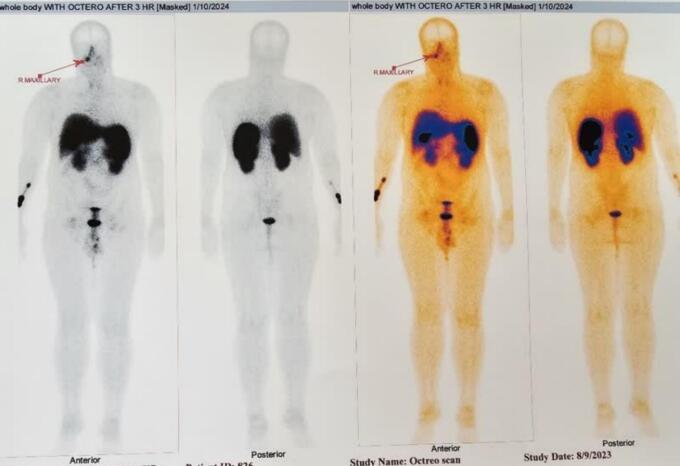
Abnormal octreotide avid lesion in right maxillary sinus.

**Fig. 3:**
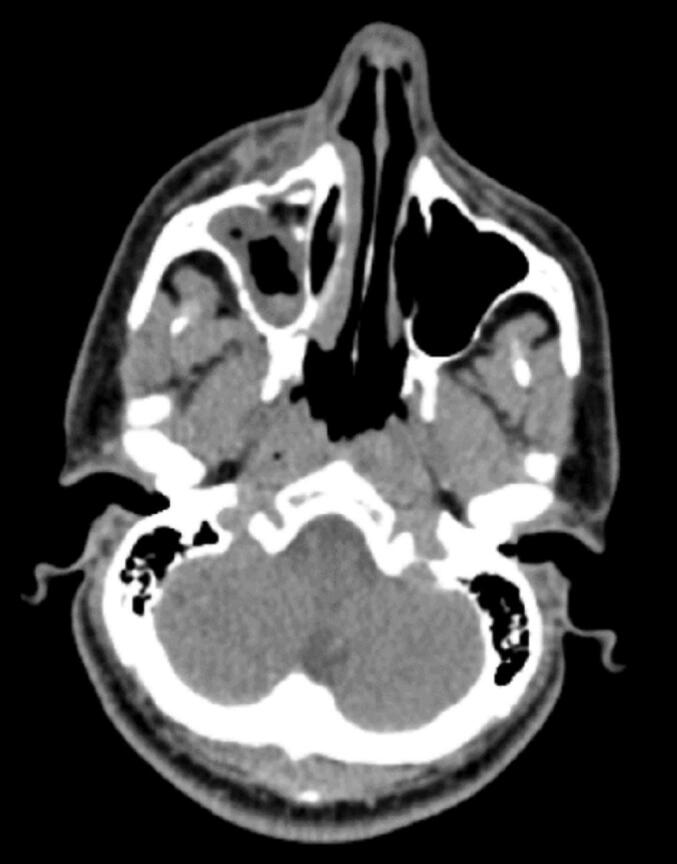
CT of Right Maxillary Sinus involvement by Tumor.

**Fig. 4:**
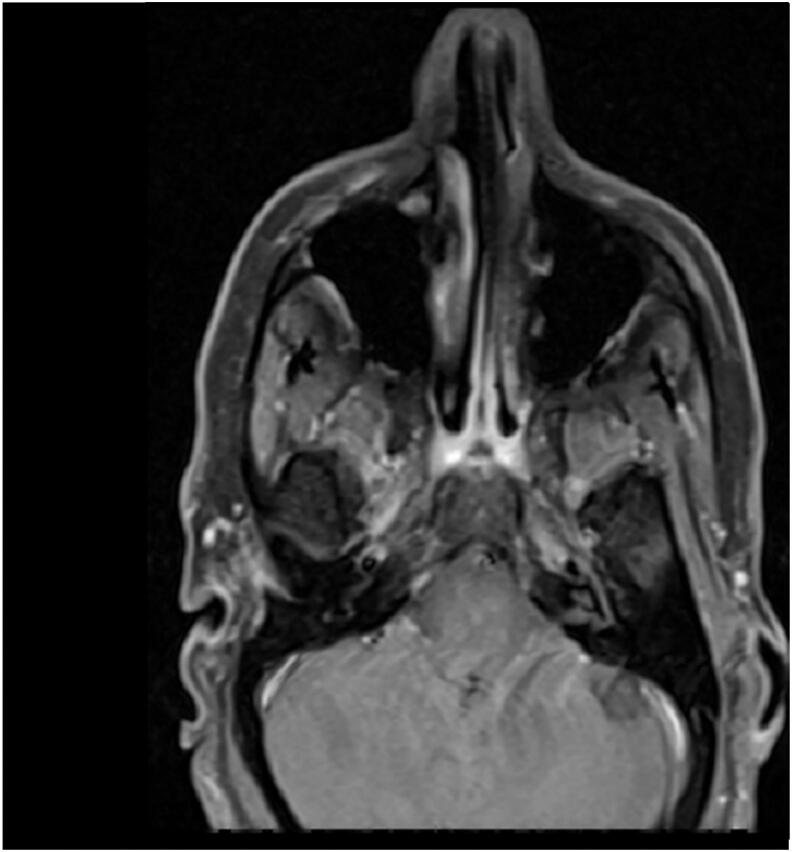
MRI of Right Maxillary sinus involved by Tumor.

Furthermore, the patient exhibited low serum phosphorus, elevated alkaline phosphatase, and high urinary phosphorus levels, indicating a potential diagnosis of osteomalacia. Given the absence of a history supporting gastrointestinal phosphorus loss, renal causes of phosphorus loss were considered for the patient. Fanconi syndrome was ruled out for the patient due to normal urine analysis. FGF-23 testing was not performed due to its unavailability. Therefore, considering the possibility of osteomalacia caused by the tumor, a whole body scan and SPECT with TC 99 octreotide were conducted. The scan revealed absorption in the right maxillary sinus (Fig. 2). Subsequently, CT scan (Fig. 3), and MRI (Fig. 4). were conducted for further investigation, confirming a neoplastic lesion in the same area with infiltration. Click here to access the complete DICOM files of the CT scan and MRI.

https://www.dicomlibrary.com?study=1.3.6.1.4.1.44316.6.102.1.2024060911243854.477156634448109352652.

https://www.dicomlibrary.com?study=1.3.6.1.4.1.44316.6.102.1.20240608235610376.83082823592345334103.

**Fig. 5:**
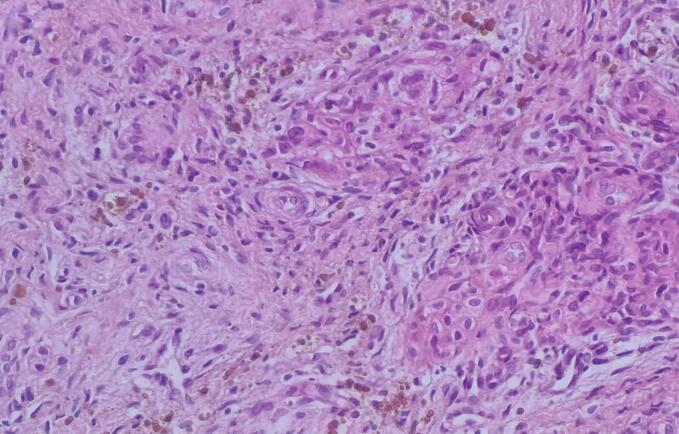
Patient Pathology of extracted tumor: Giant cell Granuloma.

A biopsy of the lesion revealed granulation tissue and significant inflammation. The patient experienced swelling at the biopsy site, promoting the administration of intravenous antibiotics as part of the treatment. Despite receiving a high daily dose of calcitriol and phosphate Sandoz, the patient's low phosphorus levels and weakness did not improve, Consequently, following the reduction of inflammation resulting from the initial biopsy, the patient underwent the removal of the lesion, which necessitated the extraction of 7 teeth. The pathology report of the removed mass revealed central giant cell granuloma (Fig. 5). After the removal of the lesion, the patient experienced a healing course and underwent one-sided fixation of the hip joint. Five months after the surgical resection, without any additional treatment, the patient's phosphorus levels and clinical condition improved compared to their state before the surgery (Table 1). Furthermore, the muscle weakness, which had previously rendered the patient bedridden and wheelchair-bound, entirely disappeared. The patient regained mobility and became able to perform daily activities.

**Table 1:** Laboratory data before and after tumor removal surgery.

	Total alkaline phosphatase	Plasma phosphate Mg/dl	Urinary phosphate Mg/dl	Plasma creatinine	Urinary creatinine Mg/dl	FEPO4	PTH	25-hydroxyvitamin D
Before tumor removal surgery	711	1.8	0.87	0.8	1	38.6%	88.4	25
After tumor removal surgery	162	3^a^	0.13	0.7	0.89	3.4%	42	27

a Without receiving any medication.

## 3 Discussion

The initial description of TIO dates back to 1974, and since then, several cases have been described. It is typically associated with mesenchymal tumors but has also been rarely observed with other malignancies such as breast cancer. In rare instances, similar to our case, tumor-induced osteomalacia has been documented to be induced by giant cell granulomas (Fernández-Cooke et al., 2015) Unlike mesenchymal tumors, giant cell granuloma are not typically classified as such; instead, they are considered reactive lesions rather than true neoplasms. It is characterized by the presence of multinucleated giant cells and is most commonly found in the jawbones (mandible and maxilla). There are two types: central giant cell granuloma (occurring within the bone) and peripheral giant cell granuloma (Saadaat et al., 2022).

Central giant cell granuloma is a rare, benign yet aggressively osteolytic tumor found in the craniomaxillofacial region. It represents <7% of all benign tumors of the jaws, with the mandible being more commonly affected than the maxilla, at a ratio ranging from 2:1 to 11:9 (Piemonte et al., 2014) While rare, there are indeed case reports of tumor-induced osteomalacia (TIO) caused by giant cell granulomas. Although giant cell granulomas are typically considered benign and reactive, there have been instances where they have been implicated in producing FGF23 and causing TIO (Fernández-Cooke et al., 2015).

It accounts for <7% of all benign tumors of the jaws, the mandible being more frequently affected than the maxilla, with a relative proportion ranging from 2:1 to 11:9. In an extensive literature review on CGCG of the jaw by de Lange, 9 none of the patients presented with In an extensive literature review on CGCG of the jaw by de Lange, 9 none of the patients presented wit If left untreated, tumor-induced osteomalacia (TIO) can be debilitating, but removal of the underlying tumor can correct the biochemical abnormality (Aligail et al., 2022). The main symptoms of the TIO are caused by the FGF23 secreted by a tumor, leading to chronic hypophosphatemia (Piemonte et al., 2014; Minisola et al., 2017; Florenzano et al., 2021). Feng et al analyzed 144 cases of TIO and found that the main signs and symptoms include skeletal discomfort, muscle fatigue, waddling gait, height reduction and disease- related fractures. All of these symptoms can significantly impact the patient's mobility (Feng et al., 2017). In some cases, depending on location and size of the lesion, the tumor may cause local symptoms. For instance, sinuses tumors might exhibit nasal congestion and bleeding (Feng et al., 2017), but these symptoms were not present in our patient. When TIO is suspected, accurately localizing the tumor is crucial. The osteomalacia symptoms can be cured by complete surgery resection (Minisola et al., 2017; Florenzano et al., 2021). Osteomalacia induced tumors are often small, slow-growing, and elusive, and can occur everywhere in the body, being in bone and soft tissue, making TIO challenging to diagnose (Cundy et al., 2020; Kumar et al., 2015).

The signs and biochemical abnormalities of TIO are frequently unclear and resemble different circumstance, for instance osteoporosis (Aligail et al., 2022). Individuals suffering from TIO are commonly misdiagnosed with osteoporosis, leading to improper therapy with antiresorptive agents like bisphosphonates (Feng et al., 2017; Brandi et al., 2021). In specific instances where the diagnosis of osteomalacia due to TIO is inconclusive—based on clinical observations, laboratory outcomes, and imaging findings— bone tissue sampling can be regarded for histological verification of osteomalacia and exclusion of other osteopathologies (Minisola et al., 2017; Minisola et al., 2023). In a study by Fernández-Cooke, which is a case report of a three-year-old child, osteomalacia was caused by a giant cell granuloma, as with our patient. However, unlike our patient, it was not possible to completely remove the lesion.

This condition must be considered in patients with osteomalacia or rickets that do not respond to treatment. If FGF23 levels are high, a tumor should be investigated as a potential cause (Fernández-Cooke et al., 2015).

## 4 Conclusion

TIO is an uncommon disorder connected with small, benign mesenchymal tumors that produce fibroblast growth factor 23 (FGF23) (Velazquez-Navarro et al., 2022). A thorough assessment of patients with osteoporosis and low serum phosphate levels should be suspected of hereditary hypophosphatemia or a cancer disease such as tumor-induced osteomalacia.

## Funding sources

Not applicable

## Ethical declaration

We obtained informed consent from the patient that we will use the patient's medical history, radiographs, CT and MRI, and pathology findings to write a case report and that the patient's name will not be published.

## CRediT authorship contribution statement

**Sevil Ghaffarzadeh Rad:** Writing – original draft, Project administration, Data curation. **Amir Bahrami:** Visualization, Methodology. **Jalil Houshyar:** Visualization, Conceptualization. **Nazila Farrin:** Writing – review & editing, Investigation. **Farzad Najafipour:** Validation, Data curation. **Halimeh Amirazad:** Writing – review & editing, Writing – original draft.

## Declaration of competing interest

No potential conflict of interest was reported by the authors.

## Data Availability

Data will be made available on request.
